# The Effects of Middle Cerebral Artery Occlusion on Central Nervous System Apoptotic Events in Normal and Diabetic Rats

**DOI:** 10.1080/15438600303727

**Published:** 2003

**Authors:** Mark Britton, Jose Rafols, Sarah Alousi, Joseph C. Dunbar

**Affiliations:** 1 Department of Physiology Wayne State University School of Medicine 5374 Scott Hall 540 E. Canfield Detroit Michigan 48201-1928 USA

## Abstract

Apoptosis and neural degeneration are characteristics of cerebral ischemia and brain damage. Diabetes is associated with worsening of brain damage following ischemic events. In this study, the authors characterize the influence of focal cerebral ischemia, induced by middle cerebral artery occlusion, on 2 indexes of apoptosis,TUNEL(terminal deoxynucleotidyl transferase–mediated deoxyuridine 5-triphosphate nick end-labeling) staining and caspase- 3 immunohistochemistry. Diabetes was induced in normal rats using streptozotocin and maintained for 5 to 6 weeks. The middle cerebral artery of both normal and diabetic rats was occluded and maintained from 24 or 48 hours. Sham-operated normal and diabetic animals served as controls. Following 24 to 48 hours of occlusion, the animals were sacrificed and the brains were removed, sectioned, and processed for TUNEL staining or caspase-3 immunohistochemistry. Middle cerebral artery occlusion in normal rats was associated with an increase in the number of both TUNEL-positive and caspase-3– positive cells in selected brain regions (hypothalamic preoptic area, piriform cortex, and parietal cortex) when compared to nonoccluded controls. Diabetic rats without occlusion showed significant increases in both TUNEL-positive and caspase-3–positive cells compared to normal controls. Middle cerebral artery occlusion in diabetic rats resulted in increases in TUNEL-positive as well as caspase-3–positive cells in selected regions, above those seen in nonoccluded diabetic rats. Both TUNEL staining and caspase-3 immunohistochemistry revealed that the number of apoptotic cells in diabetic animals tended to be greatest in the preoptic area and parietal cortex. The authors conclude that focal cerebral ischemia is associated with a significant increase in apoptosis in nondiabetic rats, and that diabetes alone or diabetes plus focal ischemia are associated with significant increases in apoptotic cells.


*Experimental Diab. Res.*, 4:13–20, 2003
Copyright © 2003 Taylor & Francis
1543-8600/03 $12.00 + .00
DOI: 10.1080/15438600390214581

### The Effects of Middle Cerebral Artery Occlusion on Central Nervous System Apoptotic Events in Normal and Diabetic Rats

**Mark Britton, Jose Rafols, Sarah Alousi, and Joseph C. Dunbar**

*Department of Physiology, Wayne State University School of Medicine, Detroit, Michigan, USA*

**Apoptosis and neural degeneration are characteristics of cerebral ischemia and brain damage. Diabetes is associated with worsening of brain damage following ischemic events. In this study, the authors characterize the influence of focal cerebral ischemia, induced by middle cerebral artery occlusion, on 2 indexes of apoptosis, TUNEL (terminal deoxynucleotidyl transferase–mediated deoxyuridine 5-triphosphate nick end-labeling) staining and caspase-3 immunohistochemistry. Diabetes was induced in normal rats using streptozotocin and maintained for 5 to 6 weeks. The middle cerebral artery of both normal and diabetic rats was occluded and maintained from 24 or 48 hours. Sham-operated normal and diabetic animals served as controls. Following 24 to 48 hours of occlusion, the animals were sacrificed and the brains were removed, sectioned, and processed for TUNEL staining or caspase-3 immunohistochemistry. Middle cerebral artery occlusion in normal rats was associated with an increase in the number of both TUNEL-positive and caspase-3–positive cells in selected brain regions (hypothalamic preoptic area, piriform cortex, and parietal cortex) when compared to nonoccluded controls. Diabetic rats without occlusion showed significant increases in both TUNEL-positive and caspase-3–positive cells compared to normal controls. Middle cerebral artery occlusion in diabetic rats resulted in increases in TUNEL-positive as well as caspase-3–positive cells in selected regions, above those seen in nonoccluded diabetic rats. Both TUNEL staining and caspase-3 immunohistochemistry revealed that the number of apoptotic cells in diabetic animals tended to be greatest in the preoptic area and parietal cortex. The authors conclude that focal cerebral ischemia is associated with a significant increase in apoptosis in nondiabetic rats, and that diabetes alone or diabetes plus focal ischemia are associated with significant increases in apoptotic cells.**

**Keywords** Apoptosis; Diabetes; Infarction; Middle Cerebral Artery

Chronic degenerative changes in the nervous system is one of the hallmarks of diabetes. Although this is a well-recognized finding, the exact mechanism of these neurological changes is not clear. Apoptosis or physiological cell death is also characteristic of diabetes and have been demonstrated in various tissues, including the cardiovascular system, the retina, and the peripheral nervous system [1–3]. The mechanism(s) associated with diabetes-related apoptosis are not defined.

The morphological expression of apoptosis is characterized by cell shrinkage, nuclear chromatin condensation, chromatin fragmentation, nuclear membrane lysis, and apoptotic body formation [4, 5]. Following ischemic brain damage, both apoptosis and neuronal degeneration are observed. In addition, apoptosis has been documented in neuropathologic changes associated with global as well as focal ischemia [6–10]. These apoptotic changes have been demonstrated to occur in a variety of brain regions, including the neocortex, striatum, and thalamus, after permanent or transient occlusion of the middle cerebral artery [11, 12]. The time sequence of apoptotic events has been characterized as beginning 6 hours after injury and remaining evident after 24 to 48 hours [13–16].

Received 4 November 2002; accepted 17 November 2002.
Address correspondence to Dr. Joseph C. Dunbar, Department of Physiology, Wayne State University School of Medicine, 5374 Scott Hall, 540 E. Canfield, Detroit, MI 48201-1928, USA. E-mail: jdunbar@ med.wayne.edu

In the present study, we examined the presence of programmed neural cell death in both normal and diabetic animals with and without cerebral ischemia using 2 methods identifying apoptotic cells, TUNEL (terminal deoxynucleotidyl transferase–mediated deoxyuridine 5-triphosphate nick end-labeling) staining and caspase-3 immunohistochemistry. The frequency of occurrence of apoptotic cells in nonlesioned control and diabetic rats was compared to control and diabetic rats subjected to occlusion of the middle cerebral artery. In addition, we evaluated selected brain regions outside of the stroke area (i.e., penumbra) to determine the spatial influence of middle cerebral artery (MCA) occlusion in normal and diabetic animals.

### MATERIALS AND METHODS

Fisher-344 rats, weighing 250 to 350 g, (Harlan, Indianapolis, IN) were used in this study. Diabetes was induced in Fisher-344 rats by injecting streptozotocin (Sigma Chemical, St. Louis, MO; dissolved in 0.1 M sodium citrate buffer, pH 4.5) at 35 to 40 mg/kg into the tail vein. The animals were maintained without any treatment for 5 to 6 weeks. They were maintained and housed according to standard guidelines. The animals had free access to rat chow and water.

The studies were conducted in 4 groups of animals: (1) nondiabetic rats without MCA occlusion; (2) nondiabetic rats with MCA occlusion; (3) diabetic rats without MCA occlusion; and (4) those with MCA occlusion.

### Focal Cerebral Ischemia

The permanent induction of selected cerebral ischemia was produced by occluding the middle cerebral artery according to the method described by Tamura and colleagues [17]. Nondiabetic and diabetic animals were anesthetized with ketamine (Fort Dodge, IA) (0.8 mg/kg intraperitoneal [IP]) and xylazine (Butler Co., Columbus, OH) (0.25 mg/kg IP). A vertical incision was made along the scalp, the temporalis muscle was divided and retracted after being exposed, and the zygomatic arch was removed. A subtemporal craniectomy was carried out using a dental drill. The left MCA was coagulated after it was located and isolated. A piece of gelfoam (UpJohn Co., Grand Rapids, MI) was used to replace the cranium. Muscles and skin were sutured in place. The animals were allowed to recover. To assess the extent of the lesions, coronal sections through the infarcted area of brains from each group were stained using Nissl methods 3 days after MCA coagulation. Twenty-four or 48 hours after MCA coagulation, the animals were used for histological studies of caspase-3 immunohistochemistry and TUNEL staining, respectively.

### Tissue Preparation for TUNEL Staining and Caspase-3 Immunohistochemistry

Five animals per group were deeply anesthetized with sodium pentobarbital (50 mg/kg) and an abdominal incision was made, with extension through the thoracic cavity in order to expose the heart. A small incision was made at the apex of the left ventricle and a catheter was inserted. Phosphate-buffered saline (PBS, 0.1 M) was administered followed by 4% paraformaldehyde. The brain was removed and coronal sections were cut with a vibratome at a thickness of 25 $\mu$m. Morphological identifications were made and quantification of in situ DNA fragmentation (TUNEL) was conducted as described previously [18]. The hypothalamic preoptic area, piriform cortex, and parietal cortex, (after Paxinos and Watson [18]) were analyzed. Within these selected areas, 500-$\mu m^2$ areas were chosen using a micrometer and the number of TUNEL-positive cells within these areas were counted.

In a parallel study, coronal brain sections from the same areas were prepared as described above. Sections were incubated with a rabbit polyclonal antibody to caspase-3 (1:400) (Cell Signaling Tech., Beverly, MA) for 1 hour. Tissue sections were then incubated with biotinylated goat anti-rabbit secondary antibody (1:500). The reaction product was visualized by 3,3-diaminobenzidine tetrahydrochloride (DAB). The sections were mounted on slides and the above described anatomical areas were analyzed and the number of caspase-3–positive cells determined as described above. Negative controls were processed by eliminating the primary antibody.

Statistical analysis of differences between the focus groups and the number of apoptotic cells within the selected areas of the brain were performed using 1-way analysis of variance (ANOVA) followed by Fisher least significant different (LSD) test.

### RESULTS

### Induction of Diabetes and Middle Cerebral Artery Occlusion

Induction of diabetes with streptozotocin resulted in a significant increase in the plasma glucose from 106 $\pm$ 3 mg/dL in normal rats to 364 $\pm$ 41 mg/dL in diabetic rats. Occlusion of the MCA did not significantly affect the glucose levels in either group. Analysis of Nissl-stained coronal sections through the region of the infarct, 72 hours after right MCA coagulation, showed degenerated neurons and reactive gliosis in ipsilateral caudate-putamen complex, frontal and dorsolateral parietal cortex, and dorsal thalamus (Figure 1*A*). No signs of obvious nerve cell degeneration or reactive gliosis were found in the regions selected for histological analyses, namely in piriform

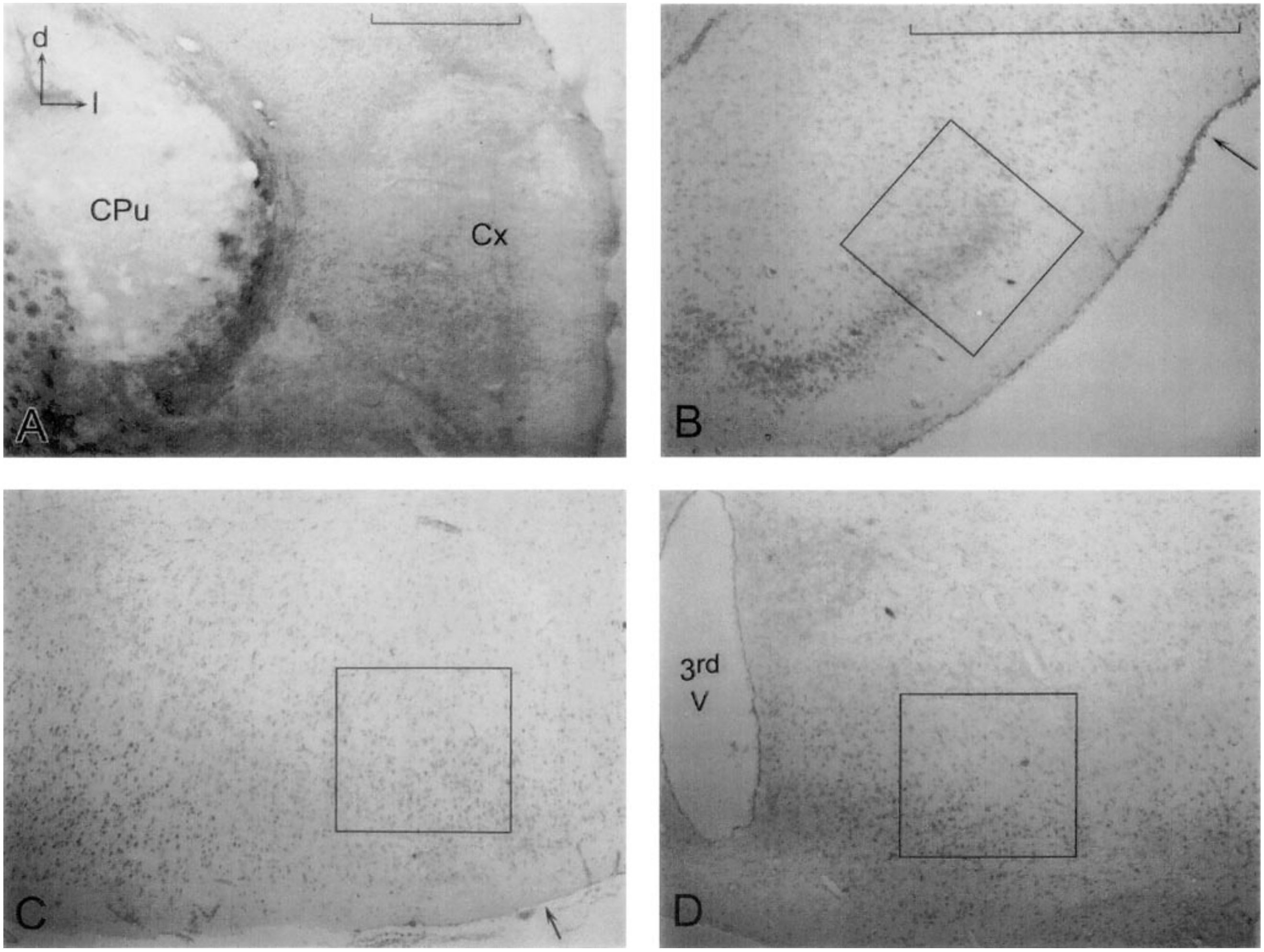

**FIGURE 1**

Low-power magnification photomicrographs of Nissl-stained sections of the rat brain illustrating the area of necrosis 72 hours after middle cerebral artery occlusion (*A*) and the three areas selected for qualitative and quantitative analyses, namely the piriform cortex (*B*), parietal cortex (*C*), and hypothalamic preoptic area (*D*). Magnification in *A* is indicated by the 1-mm scale and magnification in *B* to *D* is the same and indicated by another 1-mm scale in *B*. Orientation of *A*, *B*, and *D* is the same and indicated by the orthogonal arrows (d, dorsal and l, lateral) in *A*. In *C*, dorsal is to the right and lateral to the bottom of the micrograph. Note that the area of infarct in *A* includes both the lateral portion of the caudate-putamen complex (CPu) and adjacent parietal cortex (Cx). Single arrows in *B* and *C* point at the rhinal fissure and the pia surface, respectively. The squares in *B* to *D* outline 500-$\mu m^2$ areas that were chosen for quantitative determinations of TUNEL-positive and activated caspase-3–positive cells. The third ventricle (3rd v) in *D* is also indicated.

cortex, ventral parietal cortex (just dorsal to the rhinal fissure), and the hypothalamic preoptic area (Figure 1*B*–*D*).

### TUNEL Staining

There was a significant number of TUNEL-positive cells in all 3 areas selected for analysis in nonoccluded diabetic rats and in diabetic rats 48 hours post-MCA occlusion (Table 1). In contrast, analysis of nonoccluded normal animals revealed no TUNEL-positive cells in any of the 3 areas (Table 1). On the other hand, nondiabetic rats subjected to MCA occlusions revealed in all 3 areas significantly increased numbers of TUNEL-positive cells (Table 1), although the numbers were less than those seen in diabetic rats. Different patterns of TUNEL staining were observed depending on whether the animals were subjected to MCA occlusion alone, rendered diabetic, or a combination of both. As shown for normal piriform cortex in Figure 2, after MCA coagulation alone, TUNEL positivity was in the form of condensed nuclear chromatin in cells averaging 15 to 20 $\mu$ in diameter, which is consistent with

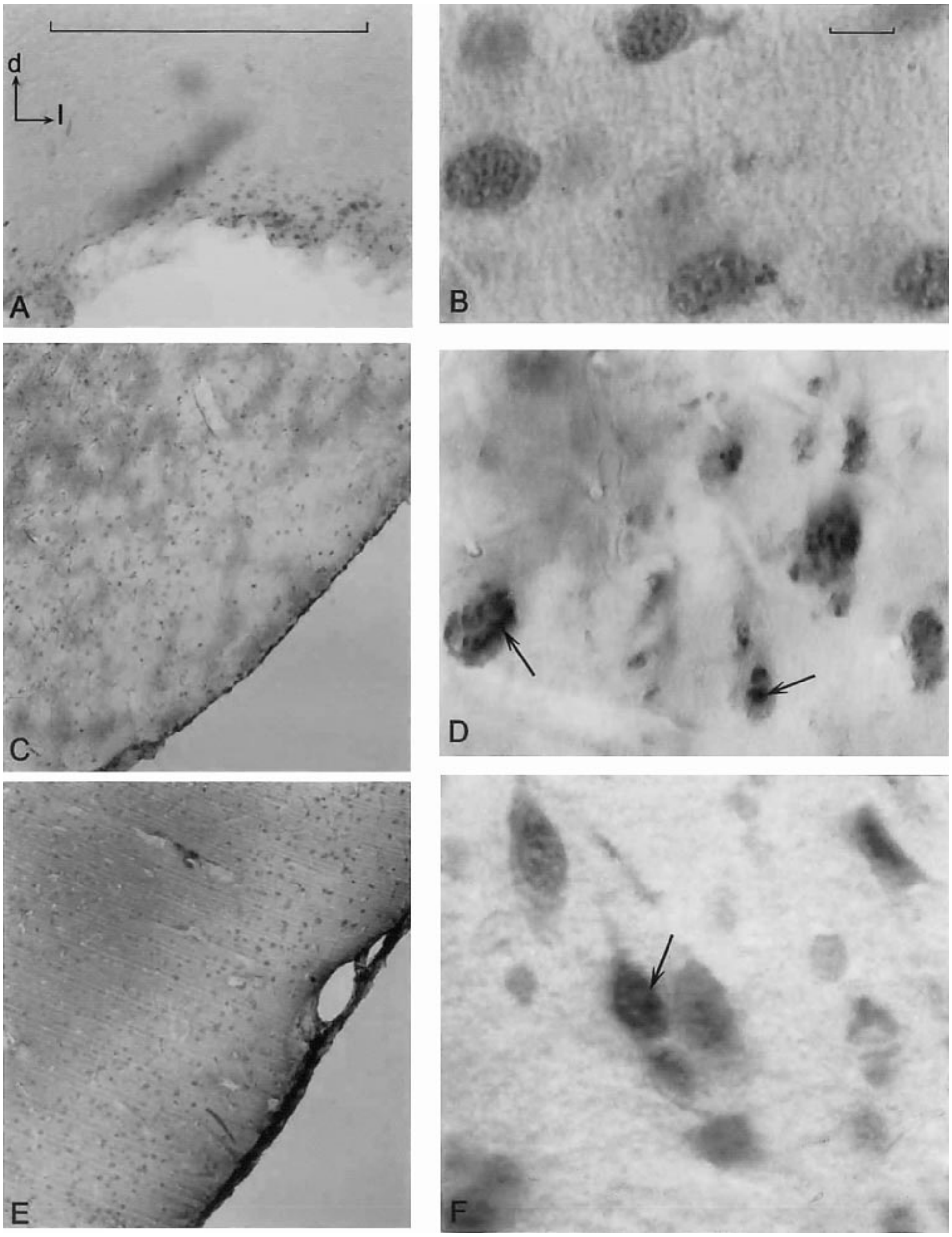

**FIGURE 2**
Paired, low/high-power photomicrographs of TUNEL staining of the right piriform cortex. *A* and *B* were stained 48 hours after a right MCA coagulation. *C* and *D* were stained 30 days after induction of diabetes without MCA coagulation. *E* and *F* were obtained after induction of diabetes (30 days) and 48 hours after stroke. Magnification in *A*, *C*, and *E* is the same and indicated by the 1-mm scale in *A*. Magnification in *B*, *D*, and *F* is indicated likewise by the 10-$\mu$ scale in *B*. Orientation of *A* to *F* is indicated by the orthogonal arrows (d, dorsal; l, lateral) in *A*. Note the condensation of the nuclear chromatin in several cells in the stroke-injured rat (*B*) and the more fragmented nature of the chromatin in the diabetic rat (*D*, *arrows*). A mixture of cells, some containing condensed chromatin and other fragmented chromatin (*D*, *arrow*) is revealed in the same diabetic plus stroke-injured rat.

**TABLE 1**
Average number of TUNEL-positive cells (per 500 $\mu m^2$) in the preoptic area and piriform and parietal cortices of normal and diabetic animals with or without occlusion

| | Normal | Normal occluded | Diabetic | Diabetic occluded |
|---|---|---|---|---|
| Preoptic area | 0.00 | $6.0 \pm 1.24^{b}$ | $17.1 \pm 6.84^{b,c}$ | $20.0 \pm 1.05^{b,c,d}$ |
| | (5) | (5) | (5) | (5) |
| Piriform cortex | 0.00 | $6.5 \pm 4.35^{b}$ | $8.5 \pm 4.17^{a,b}$ | $13.0 \pm 4.28^{a,b}$ |
| | (5) | (5) | (5) | (5) |
| Parietal cortex | 0.00 | $5.5 \pm 4.06^{b}$ | $14.6 \pm 5.42^{b}$ | $6.2 \pm 2.13^{a,b}$ |
| | (5) | (5) | (5) | (5) |

*Note.* The number in parentheses is the number of animals studied.
$^{a}P < .05$ versus preoptic diabetic and diabetic occluded; $^{b}P < .05$ versus normal; $^{c}P < .05$ versus normal occluded; $^{d}P < .05$ versus diabetic.

the size of nerve cell bodies (Figure 2*B*). A more condensed and fragmented pattern of nuclear chromatin was found in similar cells in diabetic rats (Figure 2*D*) and a mixture of both patterns was present in diabetic MCA-injured rats (Figure 2*F*). Quantification of TUNEL-positive cells in the 3 areas of analysis in diabetic rats revealed significant increases, but with wide variabilities in the number of positive cells in all 3 areas compared to those found in corresponding areas of diabetic rats subjected to MCA occlusion (Table 1). Furthermore, the number of TUNEL-positive cells was significantly increased in the preoptic area in diabetic animals following MCA occlusion when compared to diabetic animals without stroke.

### Activated Caspase-3 Staining

Although numerous caspase-3–positive cells were detected in the 3 regions of analysis of diabetic and MCA-lesioned rats, none were observed in nonlesioned normal rats. As shown for the piriform cortex, after MCA occlusion alone, caspase-3–positive cells were small (10 to 15 $\mu$) and often showed shrunken, pyknotic nuclei (Figure 3*B*, *arrowhead*). Caspase-3 positivity in the cytoplasm of the same cells was enhanced by counterstaining with hematoxilin (Figure 3*B*, *C*, *E*, *F*, *J*, *K*). The cytoplasm of cells acquired a distinctly bright orange color. In contrast, omission of hematoxylin rendered very little staining of the cytoplasm of cells, even those showing pyknotic nuclei (Figure 3*H*, *arrow*). In addition to cortex ipsilateral to the lesion, caspase-3–positive cells were found in the contralateral piriform cortex, although cells with pyknotic nuclei seemed to have little or no cytoplasmic staining (Figure 3*E*, *F*). Qualitative increases of labeled cells were seen in diabetic animals with and without MCA occlusion (Figure 3*H*, *J*, *K*) compared to normal animals (Table 2). Further quantitative analysis of the 3 selected brain areas revealed a greater number of caspase-3–positive cells in normal animals subjected to stroke alone. Additionally, the number of positive cells was increased in animals with diabetes and further increased following stroke (Table 2).

### DISCUSSION

A number of studies have demonstrated apoptosis using TUNEL-positive as well as caspase-3–positive cells as an index in a variety of different neural and neural-related tissues, such as peripheral nerves, ganglions, and the retina [1, 3, 19–22]. It is well established that the apoptotic pathway can be

**TABLE 2**
Average number of caspase-3–positive cells (per 500 $\mu m^2$) in the preoptic area and piriform and parietal cortices of normal and diabetic animals with and without occlusion

| | Normal | Normal occluded | Diabetic | Diabetic occluded |
|---|---|---|---|---|
| Preoptic area | 0.00 | $21.0 \pm 2.65^{b}$ | $4.6 \pm 6.31$ | $30.4 \pm 1.67^{b,c,d}$ |
| | (5) | (5) | (5) | (5) |
| Piriform cortex | 0.00 | $14.2 \pm 6.54^{b}$ | $7.0 \pm 7.68$ | $18.2 \pm 3.42^{b,d}$ |
| | (5) | (5) | (5) | (5) |
| Parietal cortex | 0.00 | $2.22 \pm 4.21^{b}$ | $18.8 \pm 4.32^{a,b}$ | $19.2 \pm 5.07^{a,b}$ |
| | (5) | (5) | (5) | (5) |

*Note.* The number in parentheses is the number of animals studied.
$^{a}P < .05$ versus preoptic diabetic and diabetic occluded; $^{b}P < .05$ versus normal; $^{c}P < .05$ versus normal occluded; $^{d}P < .05$ versus diabetic.

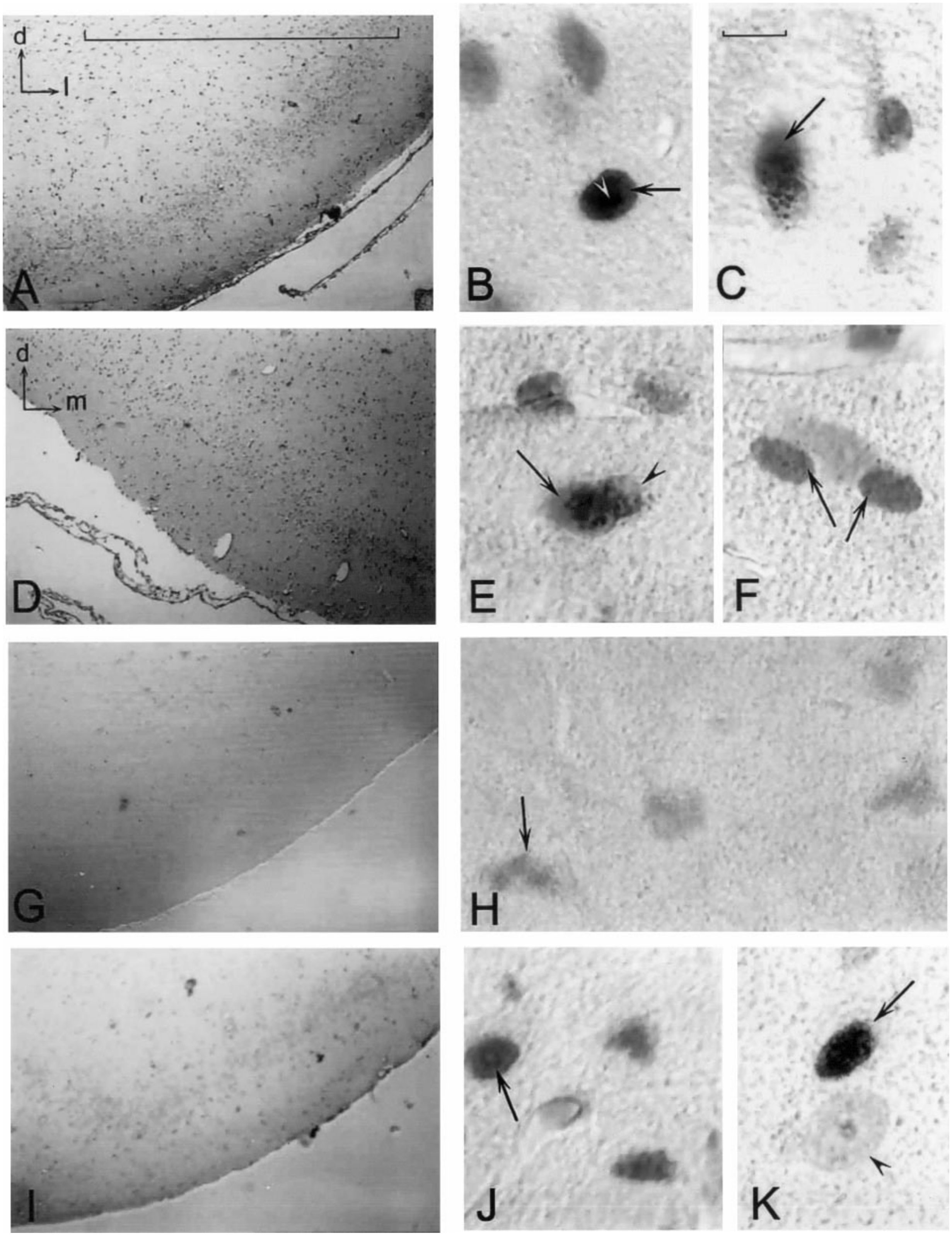

d
l
A
B
C
d
m
D
E
F
G
H
I
J
K

mediated by a number of factors, such as oxidative stress, mitochondrial dysfunction, insulin-like growth factor (IGF)-1, and insulin deficiency, all elements in the pathogenesis of diabetic complications [23]. However, caspase-3 is recognized as the final common pathway of the different apoptotic cascades and it can be activated by the membrane receptor on mitochondria signaling pathways [24]. Studies have additionally demonstrated that hyperglycemia and/or diabetes may enhance these signaling pathways or cascades, leading to an increased occurrence of apoptosis [25, 26]. In the present study, we demonstrated a significant increase in apoptosis as indexed by both TUNEL-positive and caspase-3–positive cells following the induction of focal ischemia in the forebrain of normal animals. We also demonstrated that chronic diabetes resulted in significant enhancements of the number of both TUNEL- and caspase-3–positive cells. Our observations are consistent with those of other investigators who demonstrated that diabetes as well as hyperglycemia results in worsening of brain injury after cerebral ischemia [27–29]. However, when we attempted to correlate the frequency of apoptotic cell with blood glucose levels of individual animals in this study, we observed no correlation between the magnitude of apoptosis and blood glucose levels (data not shown). Although the relationship between ischemia and diabetes is not clear, diabetes may chronically initiate apoptosis through some of the same pathways as brain injury. Diabetes causes a nutrient and metabolic deficiency syndrome and the induction of brain injury in diabetic animals may exacerbate both of these processes, resulting in further cell death.

The model we utilized in the description of ischemic brain injury is characterized as a penumbra and a core region [30–33]. These regions are described from medial to lateral on a coronal section of the brain tissue [12–14]. Anatomically, the MCA is located for the most part on the ventral aspect of the brain. Therefore, we believe that the distance of the areas that we evaluated from the MCA and its area of perfusion is another factor in characterizing brain damage caused by ischemia, especially in the focal ischemic model. When we made the anatomical correlations, it was observed that greater apoptotic frequency was seen in the hypothalamic preoptic area following focal ischemia than in uninjured control. Moreover, it was observed that in diabetic animals, there was a greater number of TUNEL-positive cells in the preoptic area but a greater number of caspase-3–positive cells in the parietal cortex. Although the distribution profiles of apoptotic cells of diabetic animals were more variable, the overall numbers were greater in diabetic animals with stroke. Both TUNEL- and caspase-3–positive cells, especially in the preoptic area of the hypothalamus and the piriform cortex, were increased to a greater extent in diabetics with focal ischemia, when compared to animals with focal ischemia alone. We do not have a good explanation for the differences in spatial distribution using the present different indexes of apoptosis in diabetic animals. One explanation may be that the preoptic area is in close proximity to the MCA and was therefore more impacted by the occlusion. In addition, the greater numbers of caspase-3–positive cells in parietal cortex may be due to the fact that the area where we conducted our analysis (i.e., just dorsal to the rhinal fissure) is the penumbral zone adjacent to the infracted core.

The identification of cells undergoing apoptosis is not entirely clear. The size of TUNEL-positive cells is consistent with the size of neurons, whereas that of caspase-3–positive (10 to 15 $\mu$) could represent shrunken neurons or even glial cells. Moreover, the finding of caspase-3–labeled cells in the piriform cortex contralateral to MCA occlusion suggests that some apoptotic cells may represent oligodedroglia associated with commissural nerve fibers damaged by the lesion.

In summary, focal ischemia using MCA occlusion increases the number of (neuronal) positive apoptotic cells. Diabetes alone results in an increased incidence of apoptosis

**FIGURE 3**

Activated caspase-3 immunostaining in the piriform cortex of the rat. The left panel consists of 4 low-power micrographs (magnification indicated by the 1-mm scale in *A*) of (*A*), ipsilateral cortex 24 hours after right MCA coagulation; (*D*) contralateral cortex 24 hours after MCA coagulation; (*G*) cortex 30 days after induction of diabetes; (*I*) ipsilateral cortex of a diabetic rat 24 hours after MCA coagulation. Except for *G* and *H*, all sections were counterstained with hematoxylin. Orientation of *A*, *G*, and *I* are indicated by the orthogonal arrows in *A* (d, dorsal; l, lateral); and that of *D* is indicated by similar arrows (d, dorsal; m, medial). The right panel includes high-power photomicrographs (*B*, *C*, *E*, *F*, *H*, *J*, *K*; 10-$\mu$ scale in *C*) taken from each of the corresponding areas on the left. After MCA coagulation, caspase-3 immunolabeling (*light orange*) occurs in the cytoplasm (*B*, *C*, *arrows*) of small 10 to 15 $\mu$) cell bodies, which also contain pyknotic nuclei (*B*, *arrowhead*). Similar findings are found in piriform cortex contralateral to MCA coagulation (*E*), although the cytoplasmic labeling in some cells appears attenuated (*F*, *arrows*). Cytoplasmic caspase-3 staining is not readily discernable when counterstaining was omitted, such as the example of the cell with pyknotic nucleus (*arrow*) from a diabetic rat (*H*). A qualitative increase in the number of caspase-3–labeled cells is observed in the diabetic rat with stroke (*J*, *K*, *arrows*). A nonlabeled neuron (*K*, *arrowhead*) is seen in the same field containing labeled cells (*arrow*).

and superimposed focal ischemia exacerbate this process. It appears that diabetes is associated with a greater sensitivity to ischemic injury in the hypothalamic preoptic area, suggesting that the enhanced incidence of apoptosis may be correlated with its distance from the MCA perfusion distribution.

### REFERENCES

[1] Barber, A., Leith, E., Khin, S., Antonetti, D., Buchanan, A., and Gardener, T. (1998) Neural apoptosis in the retina during experimental and human diabetes early onset and effect of insulin. *J. Clin. Invest.,* **102,** 783–791.

[2] Chu, Y., Farachi, F., Ooboshi, H., and Heistad, D. (1997) Increase in TUNEL positive cells in aorta from diabetic rats. *Endothelium,* **5,** 241–250.

[3] Greene, D., Stevens, M., Obrosova, I., and Feldman, E. (1999) Glucose-induced oxidative stress and programmed cell death in diabetic neuropathy. *Eur. J. Pharmacol.,* **375,** 217–223.

[4] Kerr, J. F. R., Wyllie, A. H., and Currie, A. R. (1972) Apoptosis: A basic biological phenomenon with wide ranging implication in tissue kinetics. *Br. J. Cancer,* 239–257.

[5] Majno, G., and Joris, I. (1995) Apoptosis, oncosis and necrosis: An overview of cell death. *Am. J. Pathol.,* **146,** 3–15.

[6] Heron, A., Pollard, H., Dessi, F., Moreau, J., Lasbennes, F., Ben-Ari, Y., and Charriaut-Marlangue, C. (1993) Regional variability in DNA fragmentation after global ischemia evidenced by combined histological and gel electrophoresis observations in the rat brain. *J. Neurochem.,* **61,** 1973–1976.

[7] MacManus, J. P., Buchan, A. M., Hill, I. E., Rasquinha, I., and Preston, E. (1993) Global ischemia can cause DNA fragmentation indicative of apoptosis in rat brain. *Neurosci. Lett.,* **64,** 89–92.

[8] Linnik, M. D., Miller, J. A., Sprinkle-Cavallo, J., Mason, P. J., Thompson, F. Y., Montgomery, L. R., and Schroeder, K. K. (1995) Apoptotic DNA fragmentation in the rat cerebral cortex induced by permanent middle cerebral artery occlusion. *Brain Res. Mol. Brain Res.,* **32,** 116–124.

[9] Li, Y., Chopp, M., Jiang, N., Zhang, Z. G., and Zaloga, C. (1995) Induction of DNA fragmentation after 10 to 120 minutes of focal cerebral ischemia in rats. *Stroke,* **26,** 1252–1258.

[10] Linnik, M. D., Zobrist, R. H., and Hatfield, M. D. (1993) Evidence supporting a role for programmed cell death in focal cerebral ischemia in rats. *Stroke,* **24,** 2002–2008.

[11] Garcia, J. H., Yoshida, Y., Chen, H., Li, Y., Zhang, Z. G., Lian, J., Chen, S., and Chopp, M. (1993) Progression from ischemic injury to infarct following middle cerebral artery occlusion in the rat. *Am. J. Pathol.,* **142,** 623–635.

[12] Yamada, A., Isono, M., Hori, S., Shimomura, T., and Nakano, T. (1999) Temporal and spatial profile of apoptotic cells after focal cerebral ischemia in rats. *Neurol. Med. Chir.,* **39,** 575–584.

[13] Li, Y., Chopp, M., Jiang, N., Yao, F., and Zaloga, C. (1995) Temporal profile of *in situ* DNA fragmentation after transient middle cerebral artery occlusion in the rat. *J. Cereb. Blood Flow Metab.,* **15,** 389–397.

[14] Sasaki, C., Kitagawa, H., Zhang, W., Worita, H., Sakai, K., and Abe, K. (2000) Temporal profile of cytochrome C and caspase-3 immunoactivities and TUNEL staining after permanent middle cerebral artery occlusion in rats. *Neurol. Res.,* **22,** 223–228.

[15] Tominaga, T., Kure, S., Narisawa, K., and Yoshimoto, T. (1993) Endonuclease activation following focal ischemic injury in the rat brain. *Brain Res.,* **608,** 21–26.

[16] Rupalla, K., Allegrini, P., Sauer, D., and Weissner, C. (1998) Time-course of microglia activation and apoptosis in various brain regions after permanent focal cerebral ischemia in mice. *Acta Neuropathol.,* **96,** 172–178.

[17] Tamura, A., Graham, D., McCulloch, J., and Teasdale, G. (1991) Focal cerebral ischemia in the rat: 1. Description of technique and early neuropathological consequences following middle cerebral artery occlusion. *J. Cereb. Blood Flow Metab.,* **1,** 53–60.

[18] Paxinos, G., and Watson, C. (1998) *The Rat Brain in Stereotaxic Coordinates,* 4th ed. San Diego, CA, Academic Press.

[19] Tilly, J. L. (1994) Use of the terminal transferase DNA labeling reaction for the biochemical and *in situ* analysis of apoptosis. In: *Cell Biology: A Laboratory Handbook,* Edited by Celis, J. W., vol. 1, pp. 330–337. San Diego, CA, Academic Press.

[20] Levin, L., and Louhab, A. (1996) Apoptosis of retinal ganglion cells in anterior ischemic optic neuropathy. *Arch. Ophthalmol.,* **114,** 488–491.

[21] Kerrigan, L., Zach, D., Quigley, H., Smith, S., and Pease, M. (1997) TUNEL-positive ganglion cells in human primary open-angle glaucoma. *Arch. Ophthalmol.,* **115,** 1031–1035.

[22] Thornberry, N. A., and Lazebnik, Y. (1998) Caspases: Enemies within. *Science,* **281,** 1312–1316.

[23] Guan, J., and Yankner, B. A. (2000) Apoptosis in the nervous system. *Nature,* **407,** 802–809.

[24] Hengartner, M. O. (2000) The biochemistry of apoptosis. *Nature,* **47,** 770–776.

[25] Krammer, P. H. (2000) CD95's deadly mission in the immune system. *Nature,* **47,** 789–795.

[26] Russel, J. W., Sullivan, K. A., Windebank, A. J., Herrmann, D. N., and Feldman, E. L. (1991) Neurons undergo apoptosis in animal and cell culture models of diabetes. *Neurobiol. Dis.,* **6,** 347–363.

[27] Pulsinelli, W., Levy, D., Sigsbee, B., Scherer, P., and Plum, F. (1983) Increased damage after ischemic stroke in patients with hyperglycemia with and without established diabetes mellitus. *Am. J. Med.,* **74,** 540–544.

[28] Nedergaard, M., and Diemer, N. (1987) Focal ischemia of the rat brain, with special reference to the influence of plasma glucose concentration. *Acta Neuropathol.,* **73,** 131–137.

[29] Li, P. A., Rashquinha, I., He, Q. P., Siesjo, B. K., Csiszar, K., Boyd, C., and Mac Manus, J. (2001) Hyperglycemia enhances DNA fragmentation after transient cerebral ischemia. *J. Cereb. Blood Flow Metab.,* **21,** 568–576.

[30] Astrup, J., Siesjo, B. K., and Symon, L. (1981) Thresholds in cerebral ischemia—the ischemic penumbra. *Stroke,* **12,** 723–725.

[31] Li, Y., Powers, C., Jiang, N., and Chopp, M. (1998) Intact, injured, necrotic and apoptotic cells after focal cerebral ischemia in the rat. *J. Neurol. Sci.,* **156,** 119–132.

[32] Back, T. (1998) Pathophysiology of the ischemic penumbra—revision of a concept. *Cell Mol. Neurobiol.,* **6,** 621–638.

[33] Yao, H., Takasawa, R., Fukuda, K., Shiokawa, D., Sadanaga-Akiyoshi, F., Ibayashi, S., Tanuma, S., and Uchimura, H. (2001) DNA fragmentation in ischemic core and penumbra in focal cerebral ischemia in rats. *Mol. Brain Res.,* **91,** 112–118.

